# Batzelladine D, a Marine Natural Product, Reverses the Fluconazole Resistance Phenotype Mediated by Transmembrane Transporters in *Candida albicans* and Interferes with Its Biofilm: An In Vitro and In Silico Study

**DOI:** 10.3390/md22110502

**Published:** 2024-11-05

**Authors:** Levy T. S. Domingos, Daniel C. de Moraes, Mário F. C. Santos, José A. R. Curvelo, Brayan Bayona-Pacheco, Edgar A. Marquez, Anthony W. B. Martinez, Roberto G. S. Berlinck, Antonio Ferreira-Pereira

**Affiliations:** 1 Laboratório de Bioquímica Microbiana, Departamento de Microbiologia Geral, Instituto de Microbiologia Paulo de Góes (IMPG), Universidade Federal do Rio de Janeiro, Rio de Janeiro 21941-590, RJ, Brazil; 2 Instituto de Química de São Carlos, Universidade de São Paulo, CP 780, São Carlos 13560-970, SP, Brazil; 3 Departamento de Medicina, División Ciencias de la Salud, Universidad del Norte, km 5, Vía Puerto Colombia, Área Metropolitana de Barranquilla, Barranquilla 081007, Colombia; 4 Departamento de Química y Biologia, Facultad de Ciencias Básicas, Universidad del Norte, km 5, Vía Puerto Colombia, Área Metropolitana de Barranquilla, Barranquilla 081007, Colombia

**Keywords:** batzelladine D, marine natural products, marine sponges, fluconazole resistance, multidrug transporters, anti-biofilm, *Candida albicans*, *Caenorhabditis elegans*

## Abstract

Numerous *Candida* species are responsible for fungal infections; however, *Candida albicans* stands out among the others. Treatment with fluconazole is often ineffective due to the resistance phenotype mediated by transmembrane transporters and/or biofilm formation, mechanisms of resistance commonly found in *C. albicans* strains. A previous study by our group demonstrated that batzelladine D can inhibit the Pdr5p transporter in *Saccharomyces cerevisiae*. In the present study, our aim was to investigate the efficacy of batzelladine D in inhibiting the main efflux pumps of *Candida albicans*, CaCdr1p and CaCdr2p, as well as to evaluate the effect of the compound on *C. albicans* biofilm. Assays were conducted using a clinical isolate of *Candida albicans* expressing both transporters. Additionally, to allow the study of each transporter, *S. cerevisiae* mutant strains overexpressing CaCdr1p or CaCdr2p were used. Batzelladine D was able to reverse the fluconazole resistance phenotype by acting on both transporters. The compound synergistically improved the effect of fluconazole against the clinical isolate when tested in the *Caenorhabditis elegans* animal model. Moreover, the compound disrupted the preformed biofilm. Based on the obtained data, the continuation of batzelladine D studies as a potential new antifungal agent and/or chemosensitizer in *Candida albicans* infections can be suggested.

## 1. Introduction

In recent decades, there have been significant advancements in the medical field. Nevertheless, the incidence of diseases caused by fungal pathogens continues to increase, posing a significant burden on global public health [1]. Most likely, the disease rate is even higher; however, it is masked by underreporting and underdiagnosis [2]. Fungal diseases caused by the genus *Candida*, known as candidiasis, are the most common [1]. Roughly 80% of infections are attributed to *Candida albicans*, ranging from superficial to systemic infections. The latter, frequently acquired in healthcare settings, exhibits a significantly high mortality rate, reaching up to 40% in certain countries [3].

Over the years, fluconazole (FLC) has been the primary choice for treating invasive candidiasis, but the success of this therapy has been hindered by the continuous emergence of resistant strains [4]. Resistance to fluconazole in *Candida albicans* can be multifactorial. At the same time, it is known that high-level and clinically relevant resistance is more frequently associated with the upregulation of CDR1 and CDR2. These genes encode the ATP-Binding Cassette (ABC) transmembrane transporters CaCdr1p and CaCdr2p, respectively. Both efflux pumps facilitate the extrusion of fluconazole from the cell through ATP hydrolysis, thereby impeding the drug action and consequently conferring resistance to the cell [5].

Another crucial resistance mechanism in *C. albicans* is biofilm formation. A significant number of diseases caused by *C. albicans* are linked either to biofilm development on abiotic surfaces, such as catheters, or within host tissues. Biofilms are intricate three-dimensional structures comprising cell communities surrounded by an extracellular matrix that acts as a protective barrier against the environment and provides structural integrity [6]. *Candida* biofilm resistance is multifactorial, involving various coordinated mechanisms throughout the stages of biofilm growth, including the expression of efflux pumps. As a result, the cells present in the biofilm are up to 1000 times more resistant to azoles than planktonic cells, making treatment challenging and contributing to high rates of morbidity and mortality [7].

Given the current scenario of infections caused by resistant strains of *C. albicans*, which express ABC transporter and form biofilms, the discovery of novel drugs capable of counteracting these resistance mechanisms is imperative. In a previous study, we observed that batzelladine D, a guanidine alkaloid isolated from the marine sponge *Monanchora arbuscula*, effectively reversed the fluconazole resistance phenotype in a Pdr5p-overexpressing strain of *Saccharomyces cerevisiae* [8], a transporter homologous to CaCd1p [9] and CaCdr2p [10]. Moreover, it was observed that the concentrations effective for chemosensitization are not significantly toxic for red blood cells and for a macrophage cell line [8].

Considering the homology between these transporters, as well as our previous results, the aim of this study was to investigate the effect of batzelladine D on *C. albicans* transporters CaCdr1p and CaCdr2p. Therefore, in the current study, we employed a heterologous expression system in *S. cerevisiae* strains that separately overexpresses CaCdr1p and CaCdr2p transporters [10], as well as a clinical isolate of *C. albicans* that concomitantly expresses both transporters [11]. The reversion of fluconazole resistance in the clinical isolate was also evaluated in the *Caenorhabditis elegans* animal model. We also tested the antifungal and anti-biofilm effects of batzelladine D in this context. On the other hand, aiming to gain deeper insights into the plausible mechanism and to assess the fundamental molecular interactions between batzelladine D, CaCdr1p, and CaCdr2p, a molecular docking analysis was executed. Molecular docking stands as a computational method employed to foresee the binding affinity and arrangement between minor molecules and designated target proteins. Within the realm of drug discovery, molecular docking emerges as a valuable instrument for pinpointing potential drug candidates and illuminating the operational mechanism of existing medications. In the ongoing investigation, molecular docking was harnessed to scrutinize the binding affinity between batzelladine D and the transmembrane transporters CaCdr1p and CaCdr2p present in *Candida albicans*. The outcome of this molecular docking scrutiny provides valuable insights into the interplay involving batzelladine D and these transporters, thereby facilitating the advancement of more potent antifungal agents.

## 2. Results and Discussion

### 2.1. Batzelladine D Susceptibility Test

**Figure 1:**
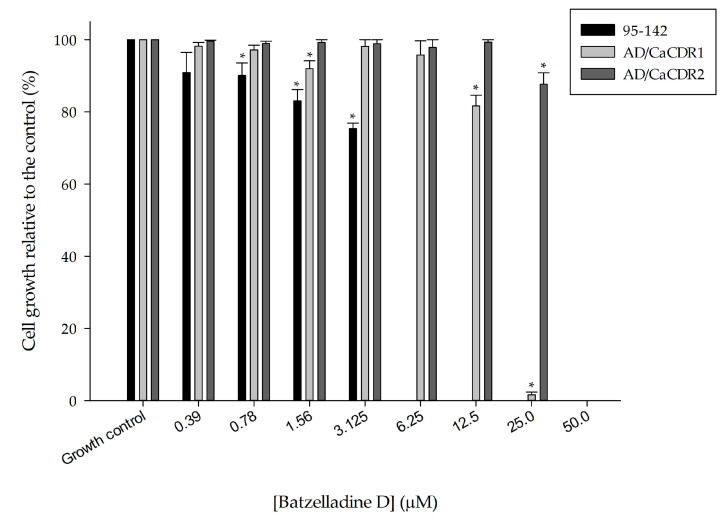
Effect of batzelladine D on the growth of 95-142, AD/CaCDR1 and AD/CaCDR2 cells. Yeast strains were incubated in the presence of two-fold serial dilutions (50–0.39 µM) of batzelladine D at 30 or 37 °C for 48 h. * Significantly lower than the untreated control (*p* < 0.05).

Batzelladine D exhibited antifungal effect against the three yeast strains tested. The strains AD/CaCDR1 and AD/CaCDR2 displayed MIC values of 25 and 50 µM, respectively. Conversely, the clinical isolate 95-142 had its growth completely inhibited at 6.25 µM (Figure 1). These results are an extension from our previous research efforts, which reported a MIC value of 25 µM of this alkaloid against the AD/PDR5+ mutant strain, known for overexpressing the endogenous transporter Pdr5p of *S. cerevisiae* [8]. The AD/PDR5+ strain was employed as the initial bioactivity screening model for batzelladine D, consistent with the approach in other studies [12,13]. In the present study, we furthered our investigation utilizing different strains featuring transporters of *C. albicans*. In this way, batzelladine D exhibited a heightened growth inhibitory effect against the clinical isolate 95-142 compared to all other strains. For the first time, batzelladine D is documented for its antifungal activity against the pathogenic yeast *C. albicans*.

### 2.2. Chemoreversing Action of Batzelladine D

**Figure 2:**
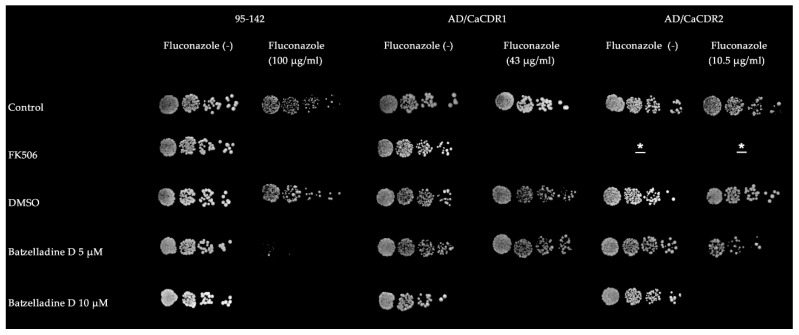
Chemosensitization of fluconazole-resistant strains by batzelladine D. Five-fold serial dilutions were spotted on YPD or Sabouraud agar in the presence or absence of subinhibitory fluconazole concentrations specific for each strain. Batzelladine D was also added to the medium at final concentrations of 5 and 10 µM. Positive chemoreversing control was conducted with FK506 at 10 µM. Negative chemoreversing controls were performed using agar medium without supplementation and DMSO 0.5%. * FK506 is not an effective inhibitor of the CaCdr2p; thus, it was not employed as a positive control for reversal on the AD/CaCDR2 strain.

Batzelladine D chemosensitized all yeast cells to fluconazole at subinhibitory concentrations (Figure 2). Both mutant strains had the fluconazole resistance phenotype completely reversed at 10 µM. On the other hand, a complete reverse effect was observed on *C. albicans* 95-142 at 5 µM. Typically, clinical isolates exhibit a lower natural efflux pump expression compared to genetically modified strains that overexpress these proteins [14]. Consequently, administering an equivalent alkaloid concentration to all three strains revealed a more pronounced chemosensitizing effect against the 95-142 strain. Previously, a complete reversing effect was also observed for the Pdr5p-overexpressing strain at 10 µM [8].

### 2.3. Interaction Between Fluconazole and Batzelladine D

The nature of interaction between fluconazole and batzelladine D was assessed using the checkerboard assay, and the resultant data are summarized in Table 1. The combination of the alkaloid with the azole drug enhanced the activity of both compounds against all tested strains. Strains 95-142 and AD/CaCDR1 exhibited FICIs ranging from 0.5 to 4.0, indicating an additive effect. Conversely, a synergistic effect was observed against the AD/CaCDR2 strain with a FICI of 0.1875. Batzelladine D reduced fluconazole MIC of 95-142, AD/CaCDR1, and AD/CaCDR2 by 2.5-fold, 4-fold, and 16-fold, respectively. The same alkaloid was synergic with fluconazole against the Pdr5p-overexpressing strain showing an 18-fold reduction in fluconazole MIC [8].

### 2.4. Molecular Docking—Proposed Interaction Between CaCdr1p and CaCdr2p with Batzelladine D

#### Redocking

Molecular docking plays a crucial role in chemical and biological studies, particularly in the field of drug discovery and design. It involves computational techniques that predict the binding modes and affinities of small molecules (ligands) to a target biomolecule (usually a protein). This method has gained immense importance due to its ability to provide insights into molecular interactions at the atomic level, which are essential for understanding biological processes and designing novel therapeutics [15]. Regarding the application of molecular docking in fungi species, Singh et al. [16] reported the use of molecular docking analyses to depict the in-silico binding affinity of geraniol with CaCdr1p in *C. albicans*. Results in this work suggest that geraniol inhibits the protein primarily by hydrophobic bonds with the residues Thr, Ser, Trp, Leu, Ile, and hydrogen bonds with the amino acids Pro and Ser, while farnesol, a known inhibitor, showed hydrophobic interactions with Arg, Glu, Thr, Asn, Lys, Glu, and Ala. No dipolar interactions were observed for this inhibitor.

After thoroughly reviewing the existing literature, it appears that no prior research has explored the molecular docking interactions of batzelladine D with CaCdr1p and CaCdr2p proteins. Given this lack of previous guidance, our study adopts a two-pronged approach: a detailed investigation of protein cavities alongside a blind docking protocol. By combining these methodologies, we aimed to generate results that closely align with experimental findings. Notably, Tacrolimus, also known as FK506 and Fujimycin, has been primarily employed as an immunosuppressive agent after organ transplantation to mitigate the risk of rejection [17]. Recent studies, however, highlight its potential as an antifungal agent, enhancing the effectiveness of azole compounds against various fungal species [18,19,20]. Similarly, milbemycin has demonstrated antifungal properties [21]. These compounds served as valuable references to gauge their efficacy against the target molecules in our investigation.

**Figure 3:**
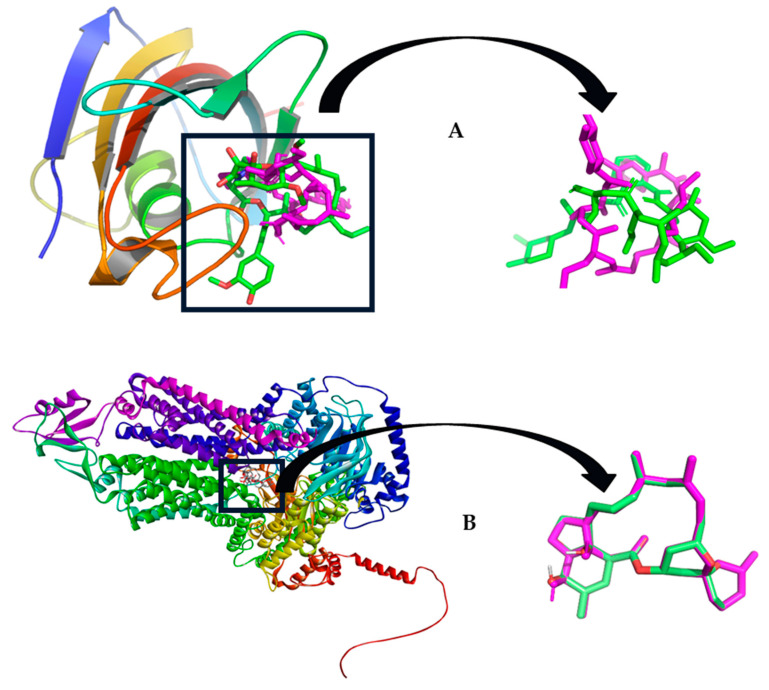
(**A**) Left: original and redocking superposition of FK506 inhibitor within the CaCdr1 protein; Right: a closer view of the superposition; (**B**) Left: Original and redocking pose superposition of milbemycin within CaCdr2 protein; Right: a closer view of the superposition.

Figure 3 shows the redocking results for both tacrolimus and milbemycin into CaCdr1p and CaCdr2p, respectively. In panel A, the left sub-figure provides a comparative display of the native configuration and the repositioned state achieved through redocking of the tacrolimus inhibitor within the spatial confines of the CaCdr1 protein. The corresponding right sub-figure offers an amplified perspective of this superposition, elucidating finer structural nuances.

Similarly, in panel B, the left sub-figure portrays a side-by-side rendition of the unaltered configuration and the redocking-induced orientation of milbemycin within the CaCdr2 protein. The complementary right sub-figure offers an enlarged portrait of the overlay, enabling a more detailed observation of the molecular congruence.

As can be observed, there is no appreciable difference between the positions of the ligands in the crystalline structure and the one obtained in redocking. Additionally, the root mean square deviation (rmsd) values obtained for tacrolimus and milbemycin were 1.008 and 0.051 Angstroms, respectively, which demonstrates that these results are reliable and can be used with a high level of accuracy.

Once the accuracy of the procedure was ensured, we proceeded to carry out molecular docking for the batzelladine D molecule with both proteins. The results are shown in Table 2. Batzelladine D exhibits scoring values of −7.5 and −8.5 kcal/mol for CaCdr1p and CaCdr2p, respectively, falling within the range indicative of non-covalent inhibition [22,23,24]; furthermore, the value obtained for CaCdr1p slightly surpasses that reported for FK506, a compound already employed as a medication against *Candida* sp. infections, and closely approaches that found for milbemycin, a reported inhibitor of CaCdr2p. In other words, the presented results significantly support the hypothesis that batzelladine D possesses the capability to inhibit these proteins, thereby augmenting the antifungal potency of fluconazole.

In our quest to demonstrate that batzelladine D could serve as a viable inhibitor for both Cacdr1p and Cacdr2p of *Candida albicans*, we conducted a docking protocol against enniatin A and beauvericin. These two compounds have been previously reported as inhibitors of CaCdr1p proteins. Specifically, enniatin A has been identified as a selective inhibitor of Cacdr1p, while beauvericin has been reported to inhibit both CaCdr1p and CaCdr2p proteins [25].

As evidenced in Table 2, the docking values mirror the pattern observed in the experimental results. For instance, enniatin A exhibits a higher scoring value for cdr1 than for cdr2, thereby demonstrating a clear selectivity towards cdr1. Similarly, beauvericin is active against both targets without any apparent selectivity. In contrast, batzelladine D displays scoring values against both proteins without any discernible selectivity. The similarity of these values with those found for beauvericin suggests that batzelladine D could potentially inhibit these two proteins.

In order to investigate the possible driving forces responsible for inhibition, it is necessary to understand the interaction between the ligand and the receptor. For this, first of all, it is necessary to ensure that the ligand does act on the active site of the receptor. In this respect, the known inhibitor serves as a guide.

**Figure 4:**
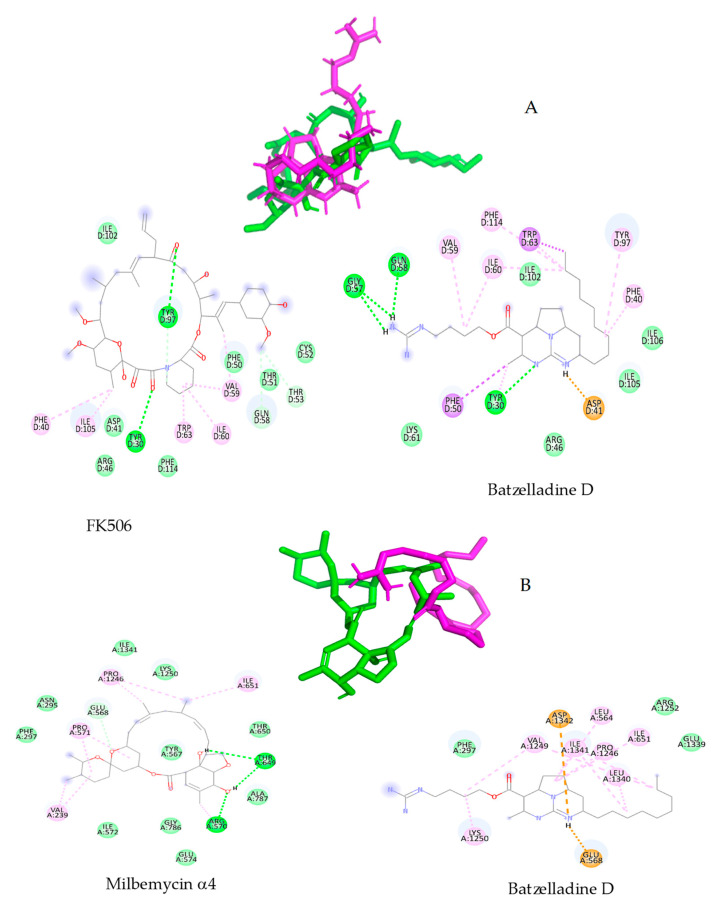
(**A**) batzelladine D-FK506 superposition into active site of CaCdr1 protein and molecular interactions diagram for FK506 and batzelladine D. (**B**) batzelladine D-milbemycin A4 superposition into active site of CaCdr2 protein and molecular interactions diagrams for both molecules.

Figure 4 shows the molecular interaction diagrams in each case. A and B represent the overlap of batzelladine D with FK506 and milbemycin A4, respectively. It is important to clarify that the overlap with rmsd > 1 Å was expected since both compounds are structurally different; FK506 and milbemycin α4 are both cyclic compounds while batzelladine D is acyclic. However, the latter is able to interact at the active site.

A detailed inspection of the molecular interaction diagrams shows an appreciable difference between FK506 and batzelladine D; according to this diagram, it could be said that FK506 interacts with CaCdr1p mainly through dipolar interactions; due to the absence of acidic hydrogens in this structure, FK506 acts as a hydrogen bond acceptor, mainly in the tyrosine residues, a fundamental residue in the inhibition as reported before [17]. Interestingly, batzelladine D shows similar interactions with the Cdr1 receptor where three strong hydrogen bonds stand out, including one bond with TyrD30, a fundamental residue for inhibition. In this case, the presence of acidic hydrogens in the structure of batzelladine D favors its participation as a hydrogen bond acceptor-donor. The difference in the scoring values can be attributed to the presence of a salt bridge type bond (coulombic interaction) between one of the nitrogens present in batzelladine D and Asn41. As is known, salt bridges represent one of the strongest non-covalent interactions [26], thus providing a higher affinity than the inhibitor FK506. Although this result has not been experimentally verified in a crystal structure, it represents additional support, suggesting that this compound could inhibit CaCdr1p and favor the antifungal activity of fluconazole.

Conversely, the interaction of milbemycin A4 with CaCdr2p closely mirrors that of FK506, predominantly driven by dipolar interactions. This interaction prominently features three hydrogen bonds involving Tyr567, Arg570, and Thr649 residues. The presence of acidic hydrogens within milbemycin A4 facilitates donor–acceptor hydrogen interactions, culminating in a scoring value exceeding 9 kcal/mol. However, in contrast to the depiction in Figure 4B, the interaction of batzelladine D with CaCdr2p primarily manifests through London interactions, albeit with two electrostatic interactions involving Asp1342 and Glu568. Consequently, the interaction energy remains below −7.0 kcal/mol, a threshold indicative of biologically active compounds [27,28,29].

In summary, both the scoring values and interaction diagrams lend additional support to the hypothesis of the potential inhibition of CaCdr proteins by batzelladine D, thereby synergistically enhancing the biological activity of fluconazole. Nevertheless, further investigations are imperative to corroborate this hypothesis.

### 2.5. Effect of Batzelladine D on Biofilm Formation

**Figure 5:**
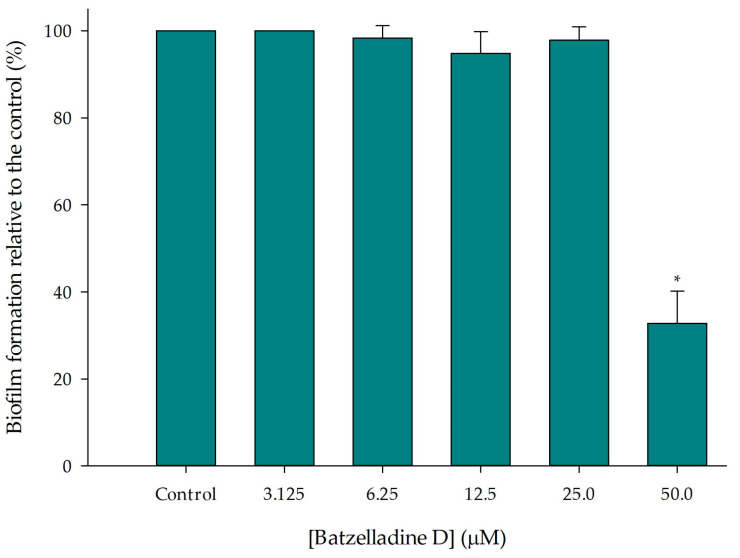
Effect of batzelladine D on *C. albicans* 95-142 biofilm formation. Yeast strains were incubated in the presence of two-fold serial dilutions (50–3.125 µM) of batzelladine D at 37 °C for 48 h. The values of the untreated control were set to 100%. * Significantly lower than the untreated control (*p* < 0.05).

The administration of batzelladine D at 50 µM resulted in a 63% reduction in the 95-142 *C. albicans* biofilm (Figure 5). However, at lower alkaloid concentrations, no significant reduction compared to the control was observed. Abd Rani and colleagues conducted a literature review on the bioactivities of batzelladine D and other fused tricyclic guanidine alkaloids. There are no reports of anti-biofilm activity in fungi [30].

### 2.6. Effect of Batzelladine D on Preformed Biofilm

**Figure 6:**
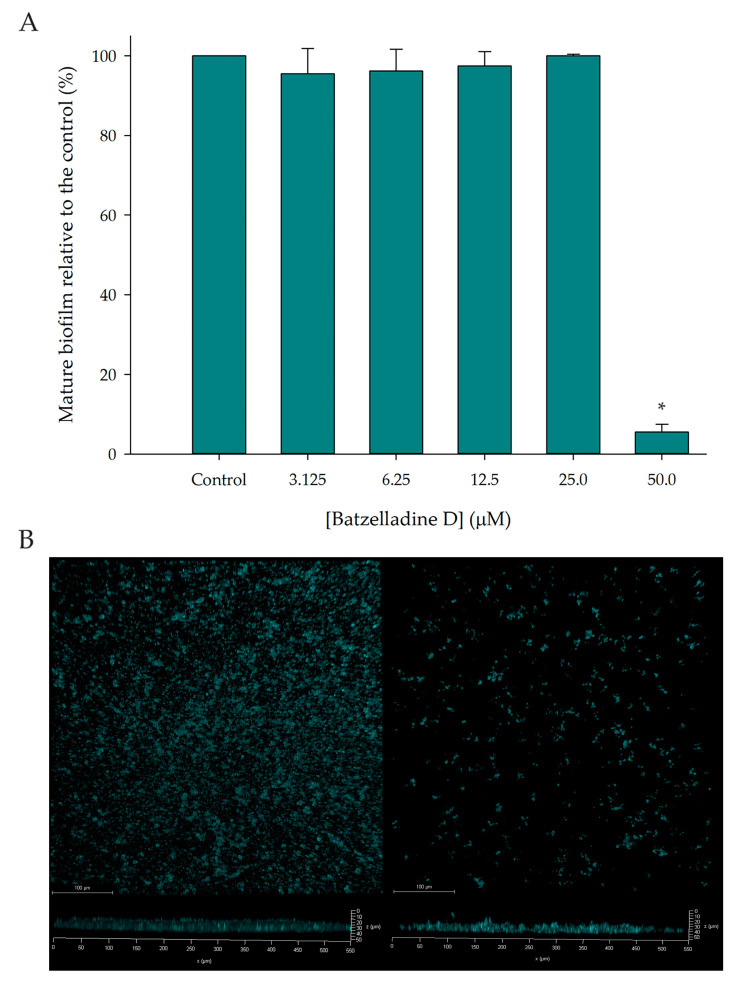
Effect of batzelladine D on *C. albicans* 95-142 preformed biofilm. (**A**) Mature biofilms were incubated at 37 °C for 24 h in the presence of two-fold serial dilutions (50–3.125 µM) of batzelladine D. The values of the untreated control were set to 100%. * Significantly lower than the untreated control (*p* < 0.05). (**B**) Confocal microscopy images of the mature biofilm control (left) and mature biofilm treated with batzelladine D 50 µM (right).

Figure 6A demonstrates a 95% inhibition of the preformed biofilm in the presence of 50 µM batzelladine D. Confocal microscopy images reveal that at this concentration, the alkaloid reduces the thickness and structure of the biofilm formed by the 95-142 strain (Figure 6B). While studies reveal upregulation of *C. albicans* efflux pump genes during biofilm formation, the azole resistance of the biofilm is intricate and not solely attributed to these proteins. Notably, even when efflux pump genes like CDR1 and CDR2 are deleted, *C. albicans* strains still form azole-resistant biofilms, highlighting the multifactorial nature of this resistance [13]. Interestingly, it was observed that batzelladine D exhibits distinct actions on *C. albicans* cells, whether they are planktonic or part of the biofilm.

### 2.7. Co-Administration of Batzelladine D and Fluconazole in Infected Caenorhabditis Elegans

**Figure 7:**
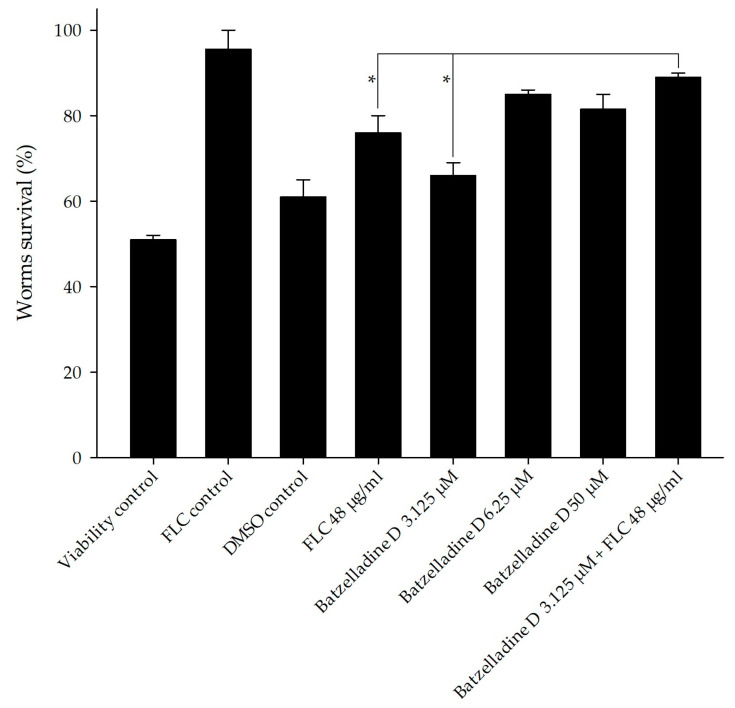
In vivo efficacy of batzelladine D and batzelladine D/fluconazole in *Caenorhabditis elegans* infected with *Candida albicans* 95-142. *C. elegans* worms were infected with *C. albicans* 95-142 and then treated with subinhibitory concentration of fluconazole (48 µg/mL); subinhibitory concentration of batzelladine D (3.125 µM); MIC value of batzelladine D (6.25 µM); effective concentration of batzelladine D against preformed biofilm (50 µM); and batzelladine D (3.125 µM) + fluconazole (48 µg/mL). Treatment with PBS (viability control), DMSO 0.5% (DMSO control), and MIC of fluconazole 128 µg/mL (FLC control) served as controls. * *p* < 0.05.

We employed the *C. elegans* model infected with the 95-142 strain to assess the impact of batzelladine D and fluconazole at concentrations predefined from previous experiments [31]. Figure 7 illustrates that all treatments involving the azole drug and the alkaloids, whether individually or in combination, were able to increase the lifespan of the worms compared to the viability control. The combined treatment, referring to the checkerboard assay results (batzelladine D 3.125 µM and fluconazole 48 µg/mL), demonstrated a significant increase in the count of live *C. elegans* compared to separate treatments at the same concentrations. Treatments using the MIC value of batzelladine D (6.25 µM), and the biofilm inhibition value (50 µM) showed a significant difference in the count of live *C. elegans* when compared to the combined treatment of batzelladine D and fluconazole. These results support the theory that the most effective treatment for this infection model is the observed additive interaction between batzelladine D and fluconazole in the checkerboard assay.

### 2.8. Batzelladine D Toxicity Test in Caenorhabditis Elegans Animal Model

**Figure 8:**
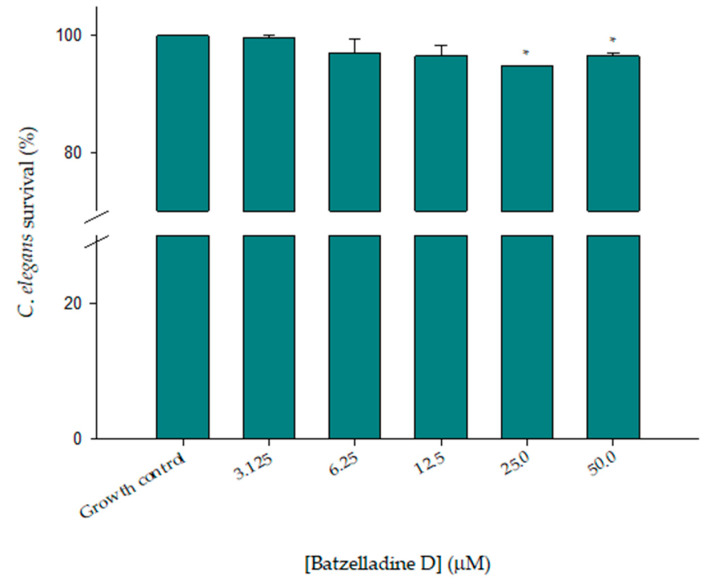
Toxicity effect of batzelladine D against *C. elegans*. Nematodes were incubated in the presence of two-fold serial dilutions (50–3.125 µM) of batzelladine D at 26 °C for 72 h, and later classified as live and dead. The values of the untreated control were set to 100%. * Significantly lower than the untreated control (*p* < 0.05).

The toxicity of batzelladine D was assessed using the *C. elegans* animal model. As depicted in Figure 8, the survival curve consistently remained above 95% across all tested concentrations of batzelladine D. Concentrations corresponding to the MIC of batzelladine D (6.25 µM) and the combined MIC (3.125 µM) were found to be non-toxic to the worm, showing no significant alteration compared to the growth control. Domingos and colleagues also noted that batzelladine D demonstrated low or non-significant toxic effects on human erythrocytes and murine macrophages [8], especially at concentrations proposed here for treating the infection caused by the 95-142 strain.

### 2.9. In Silico Toxicity Prediction

Additionally, we employed two freely available softwares for in silico assessment of batzelladine D toxicity. The results are summarized in Table 3. According to the OSIRIS Property Explorer, batzelladine D was not predicted to be mutagenic, tumorigenic, irritating or to have harmful reproductive effects, like fluconazole. Using the GUSAR software (http://www.way2drug.com/gusar/acutoxpredict.html accessed on 4 October 2023), LD_50_ values for four different administration routes were observed. The oral and subcutaneous LD_50_ values of batzelladine D are lower than those obtained for fluconazole. Conversely, the intraperitoneal and intravenous LD_50_ values are higher for batzelladine D compared to those predicted for fluconazole. The collective toxicity results support further research on batzelladine D as a potential reverser of a fluconazole resistance phenotype and/or as an antifungal agent against *C. albicans*.

## 3. Materials and Methods

### 3.1. Reagents

Fluconazole (FLC) (University pharmacy, UFJF, Juiz de Fora, Brazil) solutions were dissolved in distilled water and sterilized by filtration (0.22 µm). FK506, or tacrolimus, was purchased from Tecoland (Irvine, CA, USA). HEPES and dimethyl sulfoxide (DMSO) were purchased from Sigma-Aldrich (Sigma, St. Louis, MO, USA).

### 3.2. Isolation of Batzelladine D and Stock Solution

Batzelladine D was isolated from the marine sponge *Monanchora arbuscula*, as previously reported [32], dissolved in DMSO to a final concentration of 10 mM, and maintained at −20 °C.

### 3.3. Strains and Culture Conditions

The yeast strains used in this study are listed in Table 4. All strains were grown in YPD medium (1% yeast extract, 2% peptone, and 2% dextrose) by shaking at 100 rpm for 15 h at 30 °C (*S. cerevisiae*) or 37 °C (*C. albicans*). The experiments were performed in a range of OD_600_ 1−3, which corresponds to the exponential growth phase.

### 3.4. Susceptibility Test

The minimal inhibitory concentration (MIC) was determined by a microdilution method in a 96-well microplate according to Niimi et al. [12] with slight modifications. Yeast strains were incubated in the presence of different concentrations of batzelladine D (in a series of two-fold dilutions). Mutant *S. cerevisiae* cells were inoculated in a YPD medium at a final concentration of 1 × 10^4^ cells per well and incubated at 30 °C for 48 h with shaking (100 rpm). On the other hand, *C. albicans* cells were inoculated in an RPMI-1640 medium, according to the M27M44S protocol of the CLSI (Clinical and Laboratory Standards Institute—USA), at a final concentration of 1 × 10^3^ cells per well and incubated at 37 °C for 48 h with shaking (100 rpm). Cell growth was measured using a microplate reader at 600 nm (FLUOstar Optima, BMG Labtech, Offenburg, Germany).

### 3.5. Chemosensitization Assay—Spot Method

The chemosensitization assays, using the spot method, were performed as described previously [33]. In brief, cells were suspended to an OD_600_ of 0.1. Then, 5 µL of five-fold serial dilutions were spotted on an YPD agar (for *S. cerevisiae* strains) or Sabouraud agar (for the *C. albicans* isolate) surface in the presence or absence of subinhibitory fluconazole concentrations specific for each strain associated with batzelladine D. Controls were performed using YPD alone, YPD + 0.5% DMSO, YPD + FK506 (a classical inhibitor for CaCDR1, but not for CaCDR2). Plates were incubated at 30 °C (*S. cerevisiae*) or 37 °C (*C. albicans*) for 48 h and photographed.

### 3.6. Checkerboard Assay

The type of interaction between batzelladine D and fluconazole was evaluated according to Mukherjee et al. [34]. AD/CaCDR1 and AD/CaCDR2 cells in the exponential phase of growth were inoculated in the YPD medium at a final concentration of 1 × 10^4^ cells per well. Serial two-fold microdilutions of compounds were prepared in a 96-well microplate and then fluconazole dilutions were added to each well already containing batzelladine D; thus, each well of the plate has different combined concentrations of the azole drug and guanidine alkaloid. The microplates were incubated at 30 °C for 48 h. After incubation, the cell growth was measured using a microplate reader at 600 nm (FLUOstar Optima, BMG Labtech, Offenburg, Germany). For *C. albicans* strain, 2.5 × 10^3^ cells were inoculated per well in an RMPI-1640 medium at 37 °C for 48 h. The same dilution scheme of batzelladine D and fluconazole was implemented.

The effect of the interactions was expressed quantitatively by the fractional inhibitory concentration index (FICI). The FICI is defined as the sum of FIC (fractional inhibitory concentration) of each drug, while the FIC is the ratio MIC combined/MIC alone. The results are interpreted as follows: synergism, <0.5; indifferent or additive, 0.5–4.0; antagonism, greater than 4.0.

### 3.7. Biofilm Formation

The effect of batzelladine D on *C. albicans* biofilm formation was assessed as described by Moraes et al. [35]. Briefly, a 95-142 strain (10^7^ cells/mL) was inoculated in the wells of a 96-well polystyrene microplate containing RPMI-1640 medium and incubated for 90 min at 37 °C with agitation (75 rpm) to allow cell adhesion. Then, the supernatant was removed and the cells were washed three times with PBS to remove non-adherent cells. Thus, the cells were incubated in the RPMI-1640 medium in the presence of serial concentrations of batzelladine D (50–3.125 μM) for 48 h at 37 °C with agitation (75 rpm). Afterwards, the supernatant was removed and non-adherent cells were removed with PBS. Biofilm metabolism was evaluated by an MTT reduction assay. A solution containing 3 mg/mL MTT and 0.4 mM menadione was added to each well, and the microplate was incubated for 3 h at 37 °C in the dark. Then, a 1 h incubation with DMSO at room temperature was performed to solubilize the formazan crystals. Absorbance at 492 nm was measured using a microplate reader (Fluostar Optima, BMG Labtech, Offenburg, Germany).

### 3.8. Preformed Biofilm

The effect of batzelladine D on the preformed *C. albicans* biofilm was assessed as described by Moraes et al. [35]. Yeast cells (95-142 strain) were adhered to the wells of a 96-well polystyrene microplate as described in Section 2.7. After supernatant removal, RPMI-1640 medium was added and the cells were incubated for 24 h at 37 °C with agitation (75 rpm) to allow biofilm formation. Then, the supernatant was removed and the cells were incubated with RPMI-1640 supplemented with serial concentrations of batzelladine D (50–3.125 μM) for 24 h at 37 °C. Biofilm metabolism was quantified as described in Section 3.7.

### 3.9. Confocal Laser Scanning Microscopy (CLSM)

Biofilms in the absence or presence of 50 μM batzelladine D were formed as described in Section 2.7 and incubated with 5 μg/mL Calcofluor White for 10 min at 37 °C in the dark. Thus, biofilms were washed with PBS, analyzed by CLSM (TCS SP5, Leica, Wetzlar, Germany) at the Unidade Multiusuária de Microscopia Confocal (ICB-UFRJ, RJ, Brazil) and photographed.

### 3.10. Molecular Docking

Protein crystal structures (protein X-ray diffraction-structures, XRD) with known ligands were used in this study. Specifically, the proteins CaCdr1p and CaCdr2p proteins, associated with the ligands tacrolimus and milbemycin, respectively, were sourced from UniProt (https://www.uniprot.org) (accessed on 4 October 2023) databases, with the codes P43071 and P78595.

### 3.11. Protein and Ligand Preparations

The three-dimensional structures of both proteins, CaCdr1p and CaCdr2p, were retrieved from the UniProt database (https://www.uniprot.org/) (accessed on 4 October 2023), with the codes P43071 and P78595, respectively. After retrieval, they underwent a refinement process for proper utilization. During this phase, water molecules, solvents, and other compounds present in the crystals were removed. The resulting “clean” proteins were stored in .pdb format for subsequent integration into redocking and molecular screening procedures.

**Figure 9:**
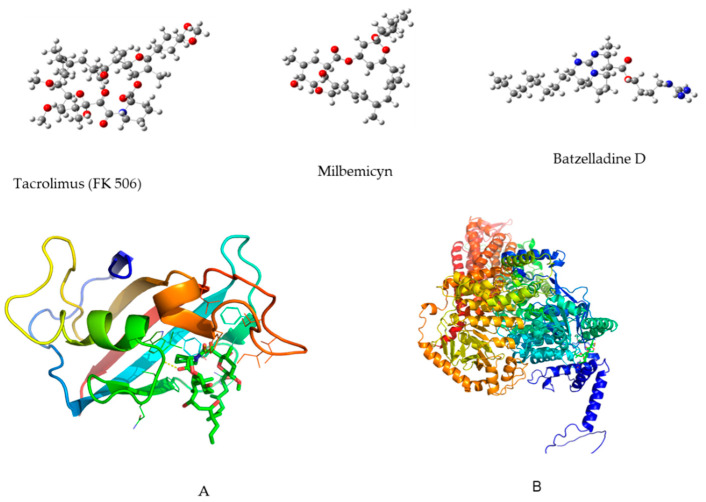
Minimum energy structure for tacrolimus, milbemicyn and batzelladine D at wB97XD/6-31G++dp level of theory. (**A**) CaCdr1p with tacrolimus as inhibitor; (**B**) CaCdr2p with milbemycin as inhibitor; both, A and B represent the structures retrieved from PDB and unitpro, respectively.

Conversely, ligand structures such as tacrolimus, milbemycin, and batzelladine D were obtained from the PubChem database (https://pubchem.ncbi.nlm.nih.gov/) (accessed on 4 October 2023). These structures were downloaded in .sdf format and subsequently subjected to theoretical optimization using the wB97XD method within the Gaussian 16 software suite for Linux. The energy-minimized structures were validated through frequency calculations, where the absence of negative frequencies ensures the presence of energy minima in each case. These structures were then transformed into .pdb format for utilization in the redocking process. Figure 9 depicts the energy-minimized structures alongside the “clean” proteins with their inhibitors.

It is important to note that in the P43071 crystal structure, the CaCdr1p structure represents a hexamer; therefore, the picture in Figure 9 represents just a monomer with a FK506 structure. On the other hand, CaCdr2 is formed by four different chains and was used as a native.

### 3.12. Redocking Protocol

The redocking procedure involved the utilization of crystal structures as illustrated in Figure 9. The ligands, specifically tacrolimus and milbemicyn, were isolated from P43071 and P78595, correspondingly. Subsequently, a meticulous redocking process was executed, returning these ligands to their initial binding sites within their respective protein environments. This intricate task was proficiently achieved using Autodock-vina, a prominent molecular docking tool renowned for its efficacy [36]. In preparation for this endeavor, Autodock tools facilitated the addition of essential polar hydrogens and flexible rotatable bonds to the ligands. Concomitantly, Kollman charges were thoughtfully incorporated into the protein structures, enhancing their accuracy and relevance. The pivotal task of defining active sites was undertaken using the Discovery study visualizer tool. For the P43071 protein, a spatial region of 26 × 26 × 26 Å was delineated, precisely centered at coordinates −16, 46, and 2 along the X, Y, and Z axes, sequentially. Meanwhile, the P-78595 protein demanded a larger bounding box, measuring 35 × 35 × 35 Å, meticulously positioned at coordinates −11, −4, and −9 along the X, Y, and Z axes, correspondingly. The robustness of this method was verified by means of root mean square deviation (RMSD). A similar protocol was employed for the batzelladine D and further, the mind molecular interactions were revealed by using a Discovery Studio Visualizer.

### 3.13. Caenorhabditis Elegans Infection Model

The AU-37 *Caenorhabditis elegans* strain was acquired from the Caenorhabidtis Genetics Center (CGC), University of Minnesota (USA) and used in this study. Worms were maintained in an NGM agar medium (3 g/L of NaCl; 17 g/L of agar; 2.5 g/L of peptone; 1 mM CaCl_2_; 5 mg/L of cholesterol in ethanol; 1 mM MgSO_4_ and 25 mM KPO_4_) at 15 °C, seeded with an *Escherichia coli* OP50 strain that was used as a food source for the nematodes.

To assess the influence of batzelladine D associated or not with fluconazole on nematode infection by *C. albicans* 95-142, the Priya’s methodology [37] was used with minor modifications, as follows. A synchronized population of nematodes was obtained by collecting eggs from pregnant adult larvae with M9 buffer (3 g of KH_2_PO_4_, 6 g of Na_2_HPO_4_, 5 g of NaCl, 1 mL of 1 M MgSO_4_ and 1 L of H_2_O) associated with 0.5 mL of 5M NaOH and 1.5 mL of 6% sodium hypochlorite. Released eggs were then centrifuged at 1300× *g* for 50 s, added to plates containing the NGM medium seeded with *E. coli* OP50 and incubated for 72 h at 25 °C to obtain synchronized larvae in stage L4 [38]. Infection of synchronized larvae with *C. albicans* 95-142 was carried out as proposed by Tempakakis et al. [39]. Briefly, 5 × 10^4^ cells of *C. albicans* 95-142 were incubated in Petri dishes containing BHI agar supplemented with 90 µg/mL of kanamycin, 200 µg/mL of streptomycin, and 200 µg/mL of ampicillin for 24 h at 37 °C. The plates were cooled to room temperature and the synchronized larvae in stage L4 were added to the plates containing the *C. albicans* seedings 95-142 and incubated for 180 min at 25 °C in order to eat the yeasts. Infected worms were then washed serially in M9 buffer and placed on clean BHI plates to crawl, aiming to remove yeast from their cuticles [40]. To evaluate the influence of batzelladine D on the nematode infection, 20 infected worms were incubated in 96-well TPP^®^ plates (Transadingem, Switzerland) containing M9 buffer with or without the batzelladine D, alone or associated with fluconazole and incubated at 25 °C for 2 days without shaking and scored as live and dead. The survival of nematodes was monitored in an inverted microscope Axiovert100 (Zeiss^®^, Jena, Germany) and determined through motility, sinus morphology and/or bulbopharyngeal movement of each helminth. The survival index was calculated from the proportion of the viable population in relation to the total number of worms including live and dead animals. This experiment was conducted independently at least twice, with a double analysis in each.

### 3.14. Batzelladine D Toxicity in Caenorhabditis Elegans Lifespan

*C. elegans* strain N2 were obtained from the Caenorhabditis Genetics Center at the University of Minnesota and handled according to the standard method [41]. Worms were maintained at 15 °C on a nematode growth medium (NGM) and routinely maintained on an *Escherichia coli* OP50 strain used as a normal diet for nematodes. Lifespan worms assay was performed as previously described with a few minor modifications [17,39]. Briefly, synchronization of worms was achieved by preparing eggs from gravid adults using a solution containing 6% NaOCl and 5M NaOH; released eggs were washed with M9 buffer and allowed to hatch overnight on NGM agar plates. Synchronized young worms were collected by washing with M9 buffer. Approximately 20 L-4 worms were added to each well of 96-well plates containing 100 mL MB medium with different concentrations of batzelladine D (two-fold serial dilutions), incubated at 26 °C for 72 h, and later classified as live and dead. The toxicity rate was calculated from the percentage of live worms out of the total number of worms, including both live and dead. Two independent experiments were conducted with a two-fold analysis in each one.

### 3.15. In Silico Toxicity Prediction

The toxicity of batzelladine D was predicted in silico by using the OSIRIS Property Explorer [41], and GUSAR (General Unrestricted Structure-Activity Relationships) [13], that provide information about toxic effects and the LD_50_ in rats, respectively.

### 3.16. Statistical Analysis

Data were analyzed using the Sigma Plot^®^ (v. 11.0) software. All the experiments were performed two or three times, and the data were expressed as mean ± standard deviation. A Student’s t-test was used for significance testing (*p*-values < 0.05 were considered significant).

## 4. Conclusions

The aim of this study was to further investigate the guanidine alkaloid batzelladine D as a chemoreverser of fluconazole resistance in yeasts. Initial screening revealed that batzelladine D effectively chemosensitized cells overexpressing endogenous transporters in *S. cerevisiae*. In the present study, we demonstrated that this effect not only extends to transporters in the pathogenic yeast *C. albicans* but is also more potent against the resistant clinical isolate 95-142, which naturally expresses the CaCdr1p and CaCdr2p transporters. Batzelladine D exhibited compelling antifungal potential against planktonic cells of *C. albicans* at 6.25 µM, along with action against biofilm formation and preformed biofilm structures. These characteristics open avenues for future studies, considering the use of batzelladine D as an antifungal agent, as well as a sanitizing and anti-biofilm agent in hospital facilities. Through the checkerboard assay, we observed an additive interaction between batzelladine D and fluconazole, allowing the use of both at concentrations below their respective MICs, achieving complete reversal of the fluconazole resistance phenotype. Interestingly, the same effect was observed in an in vivo infection model using *C. elegans*, ensuring survival for infected animals. Furthermore, the concentration of the alkaloid required for chemoreversion (3.125 µM) was non-toxic in the animal model and against other mammalian cell lines, as previously reported. An in silico study of the interaction between CaCdr1p and CaCdr2p transporters with batzelladine D suggests that the reversal effect may be due to the inhibition of these transporters, preventing the efflux of the azole drug from the cell. Further studies will be conducted to elucidate the mode of action of batzelladine D as an antifungal and chemoreverser.

## Figures and Tables

**Table 1:** Checkerboard chemosensitization assay of 95-142, AD/CaCDR1 and AD/CaCDR2 strains to fluconazole by batzelladine D.

	Batzelladine D (µM)			Fluconazole (µg/mL)				
Strains	MICa	MICb	FICc	MICa	MICb	FICc	FICId	Outcome
95-142	6.25	3.125	0.5	128	48	0.375	0.875	Additive
AD/CaCDR1	25	6.25	0.25	300	75	0.25	0.5	Additive
AD/CaCDR2	50	6.25	0.125	75	4.7	0.0625	0.1875	Synergic

MICa, MIC of compound alone; MICb, MIC of compound combined; FICc, fractional inhibitory concentration; FICId, fractional inhibitory concentration index.

**Table 2:** Scoring values (kcal/mol) obtained from autodock-vina for FK506, milbemycin A4 and batzelladine D with both, CaCdr1p and CaCdr2p, respectively. Any value represents an average from three samples.

Molecule	CaCdrp1	CaCdr2p
FK506	−8.6 ± 0.5	−7.2 ± 0.8
Milbemycin A4	−10.2 ± 0.7	−7.5 ± 0.7
Batzelladine D	−9.1 ± 0.4	−9.5 ± 0.5
Enniatin A	−9.6 ± 0.6	−7.6 ± 0.8
Beauvericin	−10.4 ± 0.3	−9.6 ± 0.5

**Table 3:** In silico prediction of batzelladine D toxicity in comparison to fluconazole by Osiris Property Explorer and GUSAR.

Osiris Property Explorer	Mutagenic	Tumorigenic	Irritant	Reproductive Effect
Batzelladine D	No	No	No	No
Fluconazole	No	No	No	No
				
GUSAR	Rat IP LD50	Rat IV LD50	Rat Oral LD50	Rat SC LD50
Batzelladine D	425.8	76.7	1342.0	1719.0
Fluconazole	708	200.4	584.4	511.3

LD50 expressed as mg/kg; IP = intraperitoneal; IV = intravenous; SC = subcutaneous.

**Table 4:** Fluconazole-resistant strains.

Species	Strain	Description	Reference
*Saccharomyces cerevisiae*	AD/CaCDR1	Mutant yeast overexpressing CDR1	[10]
*Saccharomyces cerevisiae*	AD/CaCDR2	Mutant yeast overexpressing CDR2	[10]
*Candida albicans*	95-142	FLC-resistant clinical isolate overexpressing CDR1 and CDR2	[11]

## Data Availability

All results and data were already described in this manuscript.
